# Comparison of Precision, Agreement, and Accuracy of Two Swept-Source Optical Coherence Tomography Biometers

**DOI:** 10.3390/diagnostics14212422

**Published:** 2024-10-30

**Authors:** Mercè Guarro, Meritxell Vázquez, Juan Carlos Díaz, Sergi Ruiz, Maties Gimeno, Lara Rodríguez, Elena López, Laura Sararols, Marc Biarnés

**Affiliations:** 1 OMIQ Research, 08205 Barcelona, Spain; mguarro@omiq.es (M.G.);; 2 Hospital General de Granollers, 08402 Barcelona, Spain

**Keywords:** biometry, cataract surgery, swept-source optical coherence tomography

## Abstract

**Background/Objectives**: This study’s aim was to compare the precision, agreement, and accuracy in axial length (AL) measurements of Argos^®^ (Alcon Healthcare, US) and Eyestar 900^®^ (Haag-Streit, Switzerland) swept-source optical coherence tomography (SS-OCT) biometers. **Methods**: We performed a prospective evaluation of two diagnostic devices. Three consecutive measurements of AL with the Argos^®^ and the Eyestar^®^ 900 SS-OCT biometers were conducted in random order in eyes undergoing cataract surgery in Barcelona, Spain. The main endpoint was the median difference in AL between devices. Secondary endpoints included agreement on Bland–Altman plots and 95% limits of agreement (LoAs), repeatability as measured within-subject standard deviation (S_W_), percent of failed AL measurements, percent of eyes within ±0.50 D and ±1.00 D one month after surgery, and median and mean prediction error. **Results**: We included 107 eyes of 107 patients (60.8% females, mean age of 73.1 years). The median difference in AL (Argos^®^-Eyestar 900^®^) was −0.01 mm (interquartile range [IQR], 0.06), *p* = 0.01. The 95% LoAs were −0.11 to +0.08 mm, with a trend towards less extreme measurements with Argos^®^ for very short and long eyes. The median (IQR) Sw was 0.0058 (0.0058) and 0.0000 (0.0058) for Argos^®^ and Eyestar 900^®^, respectively. There were no failed AL measurements with either device (0%, 95% CI = 0% to 3.4%). Overall, 96.1% of eyes were within ±0.50 D and 100% were within ±1.00 D. **Conclusions**: Argos^®^ and Eyestar 900^®^ provided statistically different but clinically negligible differences in AL. However, they are not interchangeable in very long or short eyes, due to the different principles used to determine AL.

## 1. Introduction

Swept-source optical coherence tomography (SS-OCT)-based biometry is increasingly used to determine axial length and other ocular parameters required for calculation of the intraocular lens (IOL) power in patients undergoing cataract surgery [1]. Its fast acquisition, precision, and safety have made them good alternatives to other approaches to determine axial length, which include ultrasound, partial interferometry, optical low-coherence reflectometry, and optical low-coherence interferometry biometers [2]. Also, its long wavelength, above 1000 nm, affords low water absorption and high tissue penetration, making SS-OCT biometers very useful in eyes with dense media opacities like mature cataracts [3], while also avoiding direct probe–eye contact that may cause patient discomfort, indentation with biased shorter-axial-length measurements, and, rarely, corneal infection.

Argos^®^ (Alcon Healthcare, Fort Worth, TX, USA) is an SS-OCT biometer that measures axial length from the cornea to the retinal pigment epithelium [2]. It uses a wavelength centered at 1060 nm, is 30 times faster than optical biometry at 3000 A-scans/s, and generates a 2D image of the eye during alignment [3]. It is connected with the Alcon Vision Planner to streamline IOL power calculations and shares information with the proprietary Verion^TM^ digital marker system for surgery planning (placing incisions, capsulotomy, etc.) and the Ora System^®^ (for intraoperative aberration measurement). It has an Enhanced Retina Visualization (ERV) module to increase 10 times the sensitivity of the detection of the posterior pole, which increases the acquisition rate of axial length measurements in cases of dense cataracts. In addition, this biometer uses segmented refractive indexes (“sum of segments”) [4] that correspond to the four actual tissues in the optical path, namely the cornea (*n* = 1.376), the aqueous humor (*n* = 1.336), the lens (*n* = 1.410), and the vitreous humor (*n* = 1.336) [4]. As such, this biometer may provide accurate axial length measurements even in eyes in which the different eye compartments have unexpectedly long or short dimensions.

The Eyestar 900^®^ (Haag-Streit, Köniz, Switzerland) is a compact SS-OCT biometer working at 1060 nm, with a scan depth range from 14 to 38 mm and a scan speed of 30,000 A-scans/s [5]. The generated 16 B-scans allow the inspection of the ocular anatomy [5]. It uses a patent-protected Mandala scan technology, which projects dense quasi-circular central and peripheral scans in the cornea that capture repeated measures at any given location, increasing the reproducibility of the measurements, which include anterior and posterior corneal topography and keratometry. The B-scans allow visualization of the anterior chamber and the lens, as well as the position and inclination of the lens, making the Eyestar 900^®^ the first biometer that can measure lens tilting [5]. The software includes a range of formulas (including the latest-generation Hill-RBF 3.0 (https://rbfcalculator.com/online/index.html, accessed on 1 September 2024)—and Olsen (https://haag-streit.com/en/products/software/eyesuite/eyesuite-iol-1/barrett-universal-ii---a-formula-for-all-seasons, accessed on 1 September 2024), analysis of aberrations, and vision simulation. In addition, the acquisition process is fully automated for both eyes, which should provide consistent measurements even with unexperienced technicians. As with all biometers (except for the Argos^®^), axial length is measured using a group refractive index [6]: a unique mean refractive index for the entire eye (*n* = 1.3375) [6]. The specifications of these two devices are summarized in Table 1.

The different approach to measure axial length (the sum of segments vs. the group refractive index) is a key difference between the Argos^®^ and Eyestar 900^®^ SS-OCT biometers, which may have clinical implications. The recent commercial availability of the Swiss device makes it possible to compare their performance in real-world practice. As such, the current study evaluated and compared the precision, agreement, and accuracy of axial length measurements of these two SS-OCT biometers, Argos^®^ and Eyestar 900^®^.

## 2. Materials and Methods

**Figure 1:**
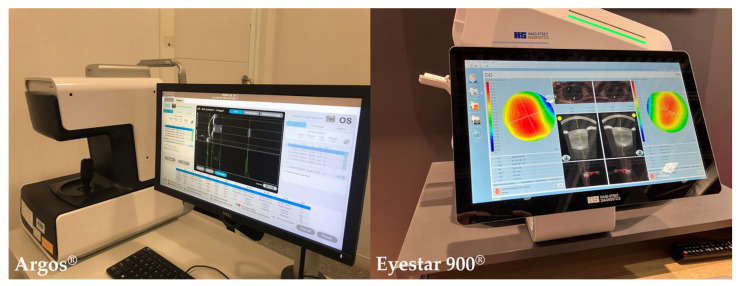
Swept-source optical coherence tomography biometers evaluated in this study. Left image, Argos^®^ (Alcon Healthcare, USA). Right image, Eyestar 900^®^ (Haag-Streit, Switzerland).

This was a prospective, longitudinal case series comparing the diagnostic performance of two SS-OCT biometers, Argos^®^ and Eyestar 900^®^ (Figure 1). This study was conducted by the OMIQ Research team in Barcelona (Spain) between October and December 2023. This study adhered to the tenets of the Declaration of Helsinki and was approved by the Quirónsalud-Catalunya Ethics Committee, and all patients signed an informed consent after explanation of the nature of this study and its potential consequences.

### 2.1. Patients

We included the first eye undergoing cataract surgery in a consecutive series of patients. Patients of any sex and race were included if they were 50 years or older and had clinically significant cataracts that required surgery due to lens opacification. Patients were excluded if they had cataract grade > 3 according to the Lens Opacities Classification System (LOCS) III [7], pre-operative corneal astigmatism ≥ 1.25 D, history of contact lens use 30 days prior to the study visit (to minimize the potential effects of lens warpage on the corneal surface), keratoconus or suspected keratoconus, severe ocular surface disease or trauma, severe retinal disease that precluded acceptable stable fixation, previous corneal refractive surgery, keratoplasty, vitrectomy or glaucoma surgery, and, for postoperative assessments, a complicated cataract surgery.

### 2.2. Procedures

All patients underwent a complete standard ophthalmic exam, which included medical history, determination of best-corrected visual acuity, intraocular pressure with a Goldman contact tonometer, anterior segment exam with a slit-lamp biomicroscopy, posterior segment exam with indirect ophthalmoscopy, and ophthalmic imaging (color fundus photography, macular or peripapilar spectral-domain optical coherence tomography, etc.) as needed. Once the indication for cataract surgery was made, patients were appointed on another day to conduct presurgical biometry.

**Figure 2:**
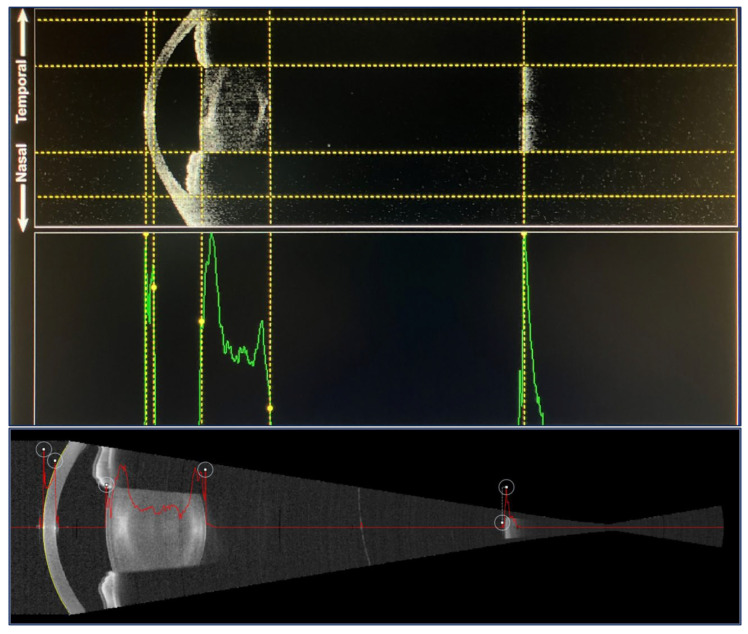
Example of swept-source optical coherence tomography B-scan biometry image of the anterior segment up to the posterior pole with each device. Top, Argos^®^ biometer. Yellow vertical lines represent the boundaries of each intraocular structure, while green lines are the intensity of the ultrasound signal in the A-scan. Bottom, Eyestar 900^®^ biometer. The red lines represent, again, the intensity of the ultrasound echo in the A-scan. In each image and from left to right, the different hyperreflective structures represent the anterior and posterior surfaces of the cornea, the anterior and posterior surfaces of the lens, and finally the retina. The hyporeflective space between the cornea and the lens represents the anterior chamber and the corresponding structure between the lens and the retina is the vitreous cavity. The group refractive index assigns a single mean n to the whole eye to derive the axial length; the sum of segments approach assigns *n* = 1.376 to the cornea, *n* = 1.336 to the aqueous and the vitreous humor, and *n* = 1.410 to the lens.

That day, three consecutive measurements of axial length were taken with each device by experienced optometrists (Figure 2). No other invasive tests (i.e., contact tonometry) or topical medications (i.e., dilating drops) were administered previously. The order of the exam with each device was randomized (using simple randomization with a random number generator), and at least 30 s was allowed between consecutive measurements. Only one eye of each patient was included; in bilateral cases, the study eye was selected at random. The Barrett Universal II formula [8] was used to determine the power of the IOL according to the measurements obtained with the Argos^®^ biometer and aiming for emmetropia in all cases.

All surgeries were performed by experienced surgeons (M.G., L.S.) using standard sutureless phacoemulsification [9]. In all cases, topical anesthesia was administered after pharmacologic mydriasis. A main clear corneal microincision of 2.2 mm was made and phacoemulsification and removal of the cataract were performed with a Centurion^®^ (Alcon, Fort Worth, TX, USA). The same model of a monofocal IOL (Clareon^®^ CNA0T0; Alcon, Fort Worth, TX, USA) was implanted in the bag in all cases through the main incision. Postoperative pharmacologic treatment included a short period with a standard combination of antibiotic and steroidal anti-inflammatory drops.

The procedure for acquiring axial length measurements with both SS OCT biometers was repeated in the study eye 4 (±2) weeks after surgery. At this visit, manifest refraction was determined by experienced optometrists (J.C.D., M.G.) following guidance from retinoscopy and/or automated refraction. These exams were followed by the standard ophthalmic exam one month after surgery.

### 2.3. Statistical Analysis

The mean (standard deviation, SD) or median (interquartile range, IQR) for quantitative and n (percentage) for categorical variables are used to describe the sample. The normality of continuous variables was assessed using the Shapiro–Wilk test.

The main endpoint was the difference in the mean of the three consecutive axial length measurements taken with the Argos^®^ and the Eyestar 900^®^ biometers using the Wilcoxon test. We also tested whether there were differences by sex. Secondary or exploratory endpoints included the agreement between devices using Bland–Altman plots and 95% limits of agreement, the within-subject standard deviation of the measurements (S_W_), and the percentage of failed axial length measurements with each biometer. Also, the mean difference between pre- and post-cataract-surgery axial length measurements with each device, the percentage of eyes with a spherical equivalent within ±0.50 D and ±1.00 D of target refraction, and the median and mean prediction errors were determined.

The required sample size was calculated according to the primary endpoint. We assumed an alpha level of 0.05, a power of 90%, a mean expected axial length of 24.10 mm [10], a mean difference of zero between both biometers with an SD of the differences of 0.30 mm and equivalence margins of ±0.10 mm (equivalent to ±0.25 D in final refraction), and an estimated required sample size to conduct the analyses of *n* = 97. Considering a 10% drop-out rate for the follow-up visit, the final sample size was *n* = 108 eyes.

All analyses were made using Stata/IC, version 15.1 (StataCorp LLC, College Station, TX, USA). A two-tailed *p*-value < 0.05 was considered statistically significant. No adjustment for multiple comparisons was made.

## 3. Results

We included 107 eyes of 107 patients with complete follow-up. Their baseline features are shown in Table 2.

**Figure 3:**
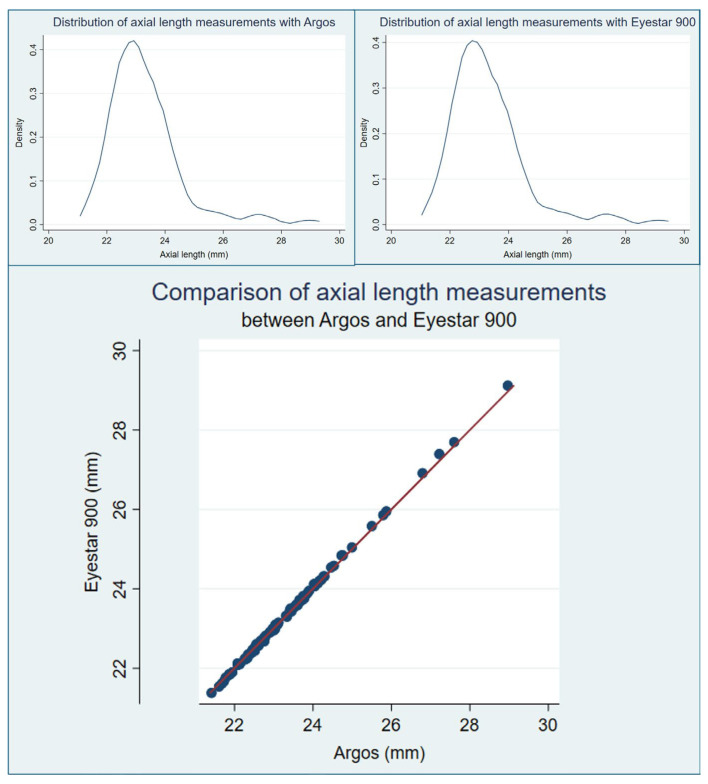
Comparison of axial length measurements between devices. Top, kernel density plots (with an Epanechnikov kernel and a bandwidth of 0.34) showing the distribution of the mean axial length measurements with Argos^®^ (left) and Eyestar 900^®^ (right). The distributions were right-skewed, leptokurtic, and virtually indistinguishable. Bottom, scatterplot comparing the values obtained with each device. Again, the values are very similar, but a slight trend towards lower values in short axial lengths and larger values in long axial lengths can be identified by slight deviation from the identity line (in red), which is more easily appreciated in the higher values.

- Primary endpoint:

The mean (SD) axial length measurement with Argos^®^ was 23.33 (1.24) mm and that with Eyestar 900^®^ was 23.35 (1.28) mm. The corresponding medians (IQR) were 23.06 (1.27) mm and 23.09 (1.29) mm for Argos^®^ and Eyestar 900^®^, respectively. The difference in the mean of the three axial length measurements with each device did not follow a normal distribution (*p* < 0.00001, Figure 3). The median difference in axial length measurements between Argos^®^ and Eyestar 900^®^ was −0.01 (IQR 0.06) mm, *p* = 0.01. The mean difference in axial length was −0.01 (SD 0.05) mm, *p* = 0.01. Therefore, the measurements with Eyestar 900^®^ were slightly larger than those of Argos^®^.

The results were similar by sex. For females, the mean (SD) axial length with Argos^®^ was 23.16 (1.36) mm and that for Eyestar 900^®^ was 23.17 (1.40) mm, while the median (IQR) was 22.91 (1.05) and 22.91 (1.10) mm, respectively. The mean difference (Argos^®^–Eyestar^®^) for females was −0.01 (SD 0.05) mm and the median was −0.01 (IQR 0.06) mm. On the other hand, the mean axial length for males with Argos^®^ was 23.54 (SD 1.05) mm and that with Eyestar 900^®^ was 23.56 (SD 1.08) mm, while the median for Argos^®^ and Eyestar 900^®^ were 23.43 (IQR 1.42) and 23.50 (IQR 1.46) mm, respectively. The mean and median difference in axial length were −0.02 (SD 0.04) and −0.02 (0.07), respectively. The differences in axial length between devices did not differ by sex (*p* = 0.06).

**Figure 4:**
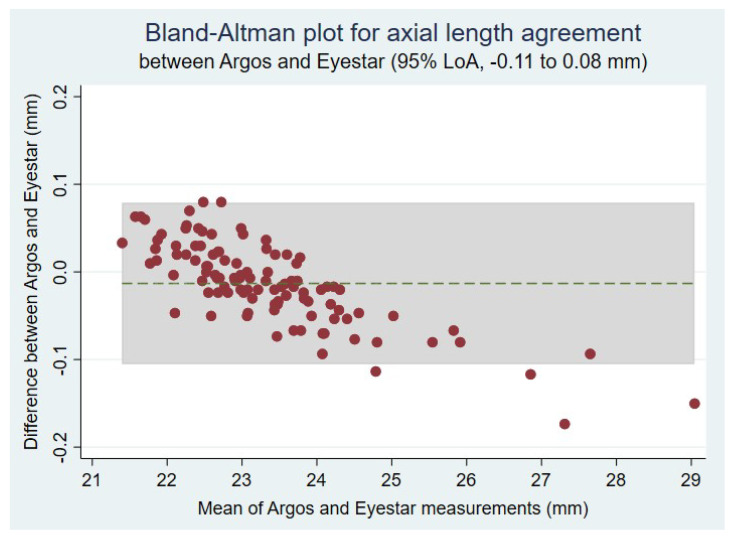
Bland–Altman plot for the agreement between Argos^®^ and Eyestar 900^®^ for axial length measurements. The descending trend implies that, for short eyes (usually associated with hyperopia), Argos^®^ provides longer axial length measurements than Eyestar 900^®^; on the other hand, for long eyes (usually associated with myopia), Eyestar 900^®^ provides longer measurements than Argos^®^. In other words, the measurements of Eyestar 900^®^ were more extreme. The trend was almost linear, and the greatest agreement was centered at approximately 23–23.5 mm. LoA: limit of agreement.

- Secondary or exploratory endpoints:

The between-instrument agreement in axial length is shown in the Bland–Altman plot in Figure 4. The results show a descending trend (with a slope of −0.030, 95% confidence interval of −0.034 to −0.025, *p* < 0.001), suggesting that measurements were more extreme with the Eyestar 900^®^ biometer: short eyes had shorter measurements and long eyes had longer axial lengths than with the Argos^®^. The 95% limits of agreement were −0.11 to 0.08 mm. The Pearson correlation coefficient between devices was 0.99 (*p* < 0.0001).

As occurred with the difference in the mean of the three axial length measurements with each device, S_W_ did not follow a normal distribution (*p* < 0.05). The median S_W_ for Argos^®^ was 0.0058 (IQR 0.0058) and that for the Eyestar 900^®^ was 0.0000 (IQR 0.0058), *p* < 0.0001. The mean S_W_ for Argos^®^ was 0.0042 (SD 0.0060) and that for the Eyestar 900^®^ was 0.0025 (SD 0.0050), *p* < 0.0001.

There were no failed axial length measurements with either device (0%). The corresponding 95% confidence intervals were 0% to 3.4%.

The median difference in axial length measurements between post- and pre-measurements for the Argos^®^ biometer was −0.01 mm (IQR 0.04), while its mean difference was −0.06 mm (SD 0.49), *p* = 0.001. For the Eyestar 900^®^, the median difference was −0.02 mm (IQR 0.05) and the mean difference was −0.03 mm (SD 0.04), *p* < 0.0001.

In terms of refractive outcomes one month after surgery (using the Argos^®^ biometer, the Barrett Universal II formula, and aiming at emmetropia), the percentage of eyes within ±0.50 D was 96.1%, and 100% were within ±1.00 D.

The prediction errors did not follow a normal distribution (*p* < 0.003). The median prediction error with Argos^®^ was 0.04 D (IQR 0.35) and that for Eyestar 900^®^ was 0.03 D (IQR 0.33), *p* = 0.08. Finally, the mean prediction error with Argos^®^ was 0.00 D (SD 0.29) and that for Eyestar 900^®^ was 0.03 D (SD 0.30), *p* = 0.05. A summary of the main findings is shown in Table 3.

Finally, no patient was excluded one month after surgery, due to intraoperative complications or adverse events in the immediate post-operative period.

## 4. Discussion

Our results suggest that in patients with cataracts not greater than grade 3 on the LOCS III scale undergoing cataract surgery, the measurements of axial length with the Argos^®^ and the Eyestar 900^®^ SS-OCT biometers are very similar. These differences are likely non-clinically relevant in standard eyes because they imply changes below 0.25 D and therefore have no refractive impact in a particular patient. Similar results have been reported for other SS-OCT biometers [11].

The median axial length measured with Argos^®^ was 0.01 (IQR 0.06) mm shorter than with Eyestar 900^®^ (*p* = 0.01). Since this difference translates into less than 0.25 D in final IOL power, they can be considered negligible. In fact, the range of their 95% limits of agreement was 0.19 mm and their correlation coefficient was 0.99. On the other hand, the agreement between both devices depends on the actual axial length, as seen on the Bland–Altman plot. The agreement is good for axial lengths between 23 and 23.5 mm, but for short eyes, Argos^®^ provides longer axial lengths, while for long eyes, Argos^®^ provides shorter measures, as compared to Eyestar 900^®^. In other words, Argos^®^ provides more contained measurements in extreme eyes. As can be seen in Figure 4, the differences between the axial length measurements obtained with each device increase progressively in the range studied (between 21.5 and 29 mm, approximately), suggesting that further departure from standard axial lengths around 23.5 mm will increase disagreement between these biometers. This was a consequence of the approach used by each device to determine axial length: increases in axial length are usually caused by an enlarged vitreous chamber, where the lower n used by the Argos^®^ as compared to the Eyestar 900^®^ (n _Vitreous_ = 1.336 vs. n _Eye mean_ = 1.3375) leads to a shorter estimation of axial length; in other words, the use of a mean refractive index of 1.3375 is too high for long eyes, where the vitreous occupies a higher proportion of the eye and the higher n used leads to overestimation of its length. This has to be taken into account because long eyes are quite prevalent. On the other hand, while short eyes (those with an axial length ≤ 22 mm) [12] are relatively less frequent, differences in axial length measurements may have a higher impact on IOL power calculation and, therefore, the final refractive outcome. Hence, these devices are not interchangeable in patients with extreme axial lengths, where the selected IOL power to achieve emmetropia may differ.

Galzignato et al. [5] showed similar results. In their study of 56 eyes, the mean axial length was 0.03 mm larger with the Eyestar 900^®^ (24.10 mm) than with Argos^®^ (24.07 mm), *p* = 0.001. The authors attributed these results to the subgroup of patients with axial lengths larger than 25 mm, whose eye dimensions were 0.08 mm larger with the Eyestar 900^®^ biometer. This difference was just 0.01 mm in eyes whose axial length was <25 mm. Probably due to the small sample size, *p*-values for these subgroups were not reported. On the other hand, in a comparison of six biometers that used different optical principles (optical low-coherence interferometry [OLCI], optical low-coherence reflectometry [OLCR], partial coherence interferometry [PCI], and SS-OCT), Montés-Micó [10] found that differences in axial length with Argos^®^ ranged from −0.07 mm (with the PCI-based AL-scan^®^) to −0.01 (with the OLCR-based Lenstar LS 900^®^). Interestingly, Argos^®^ showed the smallest range in axial length measures of all six biometers, with differences ranging from 0.04 mm (Aladdin^®^) to 0.11 mm (Lenstar LS 900^®^). Eyestar 900^®^ was not yet available when that study was published. Similar results have been reported elsewhere [2].

The segmented approach used in Argos^®^, which applies a different refraction index for the cornea, the lens, and the aqueous and vitreous cavities, may explain these discrepancies. Therefore, the absolute differences in axial length between devices when compared to biometers using this approach may depend markedly on the case-mix: if the sample is dominated by more hyperopic eyes, devices using the segmented method will tend to show longer axial lengths, and vice versa. This may be the case in the study of Montés-Micó [10]. On the other hand, given that myopic residual refraction errors tend to occur in short eyes and hyperopic outcomes are observed in long eyes [13,14,15], using a segmented approach may reduce the prediction error in these extreme eyes with most formulas, as discussed later.

The capture process was easy and fast with both biometers. In our hands, the acquisition time was faster with Eyestar 900^®^ and less demanding for the operator giving the fully automated process, but processing time was longer than with Argos^®^. Both devices provided very reproducible measurements upon repeated measurements, as determined by the low SW (<0.0060). Nonetheless, Eyestar 900^®^ was more consistent (*p* < 0.0001). The fully automated procedure may contribute to this high repeatability. These results compare favorably to those reported previously [10], which showed higher variability upon repeated measurements for biometers based on OLCI (Aladdin^®^, SW = 0.056), OLCR (Lenstar LS 900^®^, SW = 0.054), PCI (AL-Scan^®^, SW = 0.069), and other SS-OCT-based devices (IOL Master 700^®^, SW = 0.007; OA−2000^®^, SW = 0.019). In fact, these results are even better than those previously reported for Argos^®^ (SW = 0.02) [16] and Eyestar 900^®^ (SW = 0.079) in 2023 [5], which may be attributed to the study design or updates in the software of the devices.

There were no cases in which the measurements could not be obtained with either device. The high tissue penetration afforded by the long wavelength of these SS-OCT devices (1060 nm for both) allowed the determination of axial length in all cases. Biometers based on SS-OCT have been shown to provide a higher percentage rate of measurements than PCI [16] and OLCR [17] biometers. In addition to its long wavelength, Argos^®^ provides an “Enhanced Retina Visualization” (ERV) mode that increases ten times the sensitivity of the retina signal, which theoretically improves the chances to determine axial length in eyes with very significant media opacities. Tamaoki et al. [18] were unable to determine axial length in 30.5% of eyes with dense cataracts (grades ≥ 4 on the Emery–Litle classification) using the standard mode of Argos^®^ and in 38.5% with IOLMaster 700^®^ (*p* = 0.083) [18]. Nonetheless, 78.5% of these eyes could be measured when the ERV mode was used [18]. Given the frequency of dense cataracts (LOCS III > 3), especially in low-income countries where the coverage of cataract surgery is lower [19], this is an important topic. While SS-OCT biometers are becoming the reference standard for pre-surgical biometry [20], a comparison of failure rates between these devices is a relevant endpoint to further characterize their performance.

The median axial length measurement before and after surgery differed slightly, being 0.01 mm and 0.02 mm shorter after surgery for Argos^®^ and Eyestar 900^®^ (*p* = 0.001 and *p* < 0.0001, respectively). Albeit statistically significant, these differences are not clinically relevant and compare favorably to those reported in the literature [18], suggesting that measurements were unaffected by the degree of media opacities before surgery.

The refractive outcomes were excellent when targeting for emmetropia using either the Argos^®^ or the Eyestar 900^®^ axial lengths imputed using one of the most accurate formulas in different studies [21,22,23], the Barrett Universal II: more than 95% of eyes were within ±0.50 D and 100% were within ±1.00 D one month after surgery. Also, the median prediction errors were 0.04 D and 0.03 D with Argos^®^ and Eyestar 900^®^, respectively. These results confirm the excellent predictability of measurements derived from both devices in standard eyes who have not undergone previous corneal refractive surgery, and agree with mean prediction errors reported with SS-OCT biometers by Multack et al. [24] (0.07 D [SD 0.32] for Argos^®^ and 0.08 [SD 0.34] for IOLMaster 700^®^), Shammas et al. [25] (0.00 D [SD 0.39] with Argos^®^), An et al. [26] (0.00 D [SD 0.44] with Argos^®^), and Sorkin et al. [27] (0.01 D [SD 0.55] with Eyestar 900^®^ using reflective keratometry to estimate corneal curvature, and 0.07 D [0.62] using the same device with the keratometry derived from the SS-OCT). While the differences in prediction error when using the segmented approach were negligible in standard eyes, highly hyperopic or myopic eyes may benefit from this method. In fact, Shammas et al. [25] and Blehm et al. [28] reported incrementally improved results in eyes with very long or short axial length measurements. Therefore, in settings with a marked prevalence of high or pathologic myopia [29,30] (Asian regions, tertiary retinal clinics) or high hyperopia [31,32] (pediatric patients, again specialized retina sites), the difference between devices using the groups’ refractive index or the sum of segments approach may become clinically relevant. These studies should focus on endpoints that are relevant to the patient, like percentage of eyes within +0.50 D or ±1.00 D or median absolute error, rather than mean or median axial length comparisons. A proper interpretation of future studies should account for the influence of these factors in the results.

The main limitation of this study is the relatively small number of eyes with very long or very short axial length. While this study prospectively enrolled a consecutive case series of patients undergoing cataract surgery and therefore is highly representative of most cases in the clinical setting, the difference in axial length measurements observed in eyes with high myopia and hyperopia warrants further scrutiny. We did not attempt to evaluate these subgroups separately, due to the small sample size. Future studies may focus on the characterization of prediction error of SS-OCT biometers based on the sum of segments or the group refractive index strategies in eyes with extreme axial lengths, and the determination of potential correction formulas that minimize residual refractive error. Additionally, the performance of SS-OCT devices in patients with cataracts grade LOCS III ≥ 4 should be evaluated, both in terms of acquisition rate and, importantly, in the precision of measurements as determined by prediction error and residual refractive error after surgery.

## 5. Conclusions

In summary, there were statistically significant differences in axial length measurements between the Argos^®^ and the Eyestar 900^®^ SS-OCT biometers, but these were clinically negligible. Axial length was obtained in all eyes in which it was attempted with both devices, and refractive outcomes were similar when considering the IOL power derived from their measurements. Nonetheless, they may not be interchangeable in extreme eyes, due to the different approaches used to determine axial length.

## Figures and Tables

**Table 1:** Summary of technical characteristics of Argos^®^ and Eyestar 900^®^ SS-OCT biometers. AL: axial length: N/R: not reported by the manufacturer.

Characteristics	Argos^®^	Eyestar 900^®^
Manufacturer	Alcon Healthcare	Haag-Streit
Method	SS-OCT	SS-OCT
Center wavelength	1060 nm	1060 nm
Scan speed	3000 A-scans/s	30,000 A-scans/s
B-scan axial resolution	50 µm	N/R
Scan depth range	50 mm	14 to 38 mm
Last generation formulas available	Yes	Yes
AL measurement approach	Sum of segments	Group refractive index
Other	Enhanced Retina Visualization, integration with other devices	Quantification of lens tilting, vision simulator

**Table 2:** Baseline features of participants in this study. Values represent mean (standard deviation) for quantitative variables and n (percentage) for categorical variables.

Characteristics	Value
N, eyes	107
Age	73.1 (7.8) years
Females	65 (60.8%)
Right eyes	51 (47.7%)

**Table 3:** Summary of results for the Argos^®^ and Eyestar 900^®^ SS-OCT biometers measurements. Differences in pre- and post-AL refer to changes in AL measured after surgery. Values represent the median (interquartile range) for quantitative variables (except where noted with *, where they represent mean [standard deviation]) and n (percentage) for categorical variables. AL: axial length; corr. coeff.: correlation coefficient; LoA: limit of agreement; N/A: does not apply; S_W_: within-subject standard deviation.

Parameter	Argos^®^	Eyestar 900^®^	*p*-Value
AL *	23.33 (1.24) mm	23.35 (1.28) mm	<0.0001
95% LoA	−11 to 0.08 mm		N/A
Pearson corr. coeff.	0.99		<0.0001
S_W_	0.0058 (0.0058)	0.0000 (0.0058)	<0.0001
Failed AL measurements	0 (0.00%)	0 (0.00%)	1.00
Differences in pre-and post-AL	−0.01 (0.04) mm	−0.02 (0.05) mm	N/A
Prediction error	0.04 (0.35) D	0.03 (0.33) D	0.08

## Data Availability

Data may be available upon reasonable request and after approval by the sponsor.
